# The utility of fat mass index vs. body mass index and percentage of body fat in the screening of metabolic syndrome

**DOI:** 10.1186/1471-2458-13-629

**Published:** 2013-07-03

**Authors:** Pengju Liu, Fang Ma, Huiping Lou, Yanping Liu

**Affiliations:** 1 Department of Clinical Nutrition, Peking Union Medical College Hospital, China Academic Medical Science and Peking Union Medical College, Beijing 100730, China; 2 Department of Medical examination center, Peking Union Medical College Hospital, China Academic Medical Science and Peking Union Medical College, Beijing 100730, China; 3 No.1 ShuaiFuyuan, Wang Fujing ST, Dongcheng District, Beijing 100730, China

**Keywords:** Metabolic syndrome X, Body composition, Fat mass index, Body mass index, Percentage of body fat, Screening

## Abstract

**Background:**

It has been well documented that obesity is closely associated with metabolic syndrome (MetS). Although body mass index (BMI) is the most frequently used method to assess overweightness and obesity, this method has been criticized because BMI does not always reflect true body fatness, which may be better evaluated by assessment of body fat and fat-free mass. The objective of this study was to investigate the best indicator to predict the presence of MetS among fat mass index, BMI and percentage of body fat (BF %) and determine its optimal cut-off value in the screening of MetS in practice.

**Methods:**

A cross-sectional study of 1698 subjects (aged 20–79 years) who participated in the annual health check-ups was employed. Body composition was measured by bioelectrical impedance analysis (BIA). Fat mass index (FMI) was calculated. Sex-specific FMI quartiles were defined as follows: Q1: <4.39, Q2:4.39- < 5.65, Q3:5.65- < 7.03, Q4:≥7.03,in men; and Q1:<5.25, Q2:5.25- < 6.33, Q3:6.33- < 7.93,Q4:≥7.93, in women. MetS was defined by National Cholesterol Education Program/Adult Treatment Panel III criteria. The association between FMI quartiles and MetS was assessed using Binary logistic regression. Receiver operating curve(ROC) analysis was used to determine optimal cutoff points for BMI,BF% and FMI in relation to the area under the curve(AUC),sensitivity and specificity in men and women.

**Results:**

The adjusted odds ratios (95% CI) for the presence of MetS in the highest FMI quartile versus lowest quartile were 79.143(21.243-294.852) for men( *P* < 0.01) and 52.039(4.144-653.436) for women( *P* < 0.01) after adjusting age, BMI, BF%, TC, LDL, CRP, smoking status and exercise status, and the odds ratios were 9.166(2.157-38.952) for men( *P* < 0.01) and 25.574(1.945-336.228) for women( *P* < 0.05) when WC was also added into the adjustment. It was determined that BMI values of 27.45 and 23.85 kg/m^2^, BF% of 23.95% and 31.35% and FMI of 7.00 and 7.90 kg/m^2^ were the optimal cutoff values to predict the presence of MetS among men and women according to the ROC curve analysis. Among the indicators used to predict MetS, FMI was the index that showed the greatest area under the ROC curve in both sexes.

**Conclusions:**

Higher FMI levels appear to be independently and positively associated with the presence of MetS regardless of BMI and BF%. FMI seems to be a better screening tool in prediction of the presence of metabolic syndrome than BMI and percentage of body fat in men and women.

## Background

Obesity is one of basic clinical conditions of metabolic syndrome (MetS), which is a cluster of risk factors for cardiovascular disease (CVD). The clustering of factors includes overweight/obesity, hyperinsulinemia, hypertension, hyperlipidemia, fasting hyperglucose or type 2 diabetes mellitus, and obesity (particularly central obesity) plays a central role in the MetS [1]. The growing prevalence of overweight and obesity are established risk factors for the metabolic syndrome [2].

Although body mass index (BMI) is the most frequently used method to assess overweightness and obesity, this method has been criticized because BMI does not always reflect true body fatness [3-6] and has some limitations in assessing the risk of obesity-related diseases in persons with low muscle and high body fat [7], and in individuals with increased body fat and normal BMI. A recent study [8] reported that normal weight obesity was associated with MetS and insulin resistance,and suggested that clinical assessment of excess body fat in normal-BMI individuals should begin early in life. It seems that true body fatness may be better evaluated by assessment of body fat and fat-free mass [9]. Therefore,much research has recently examined the potential role of body composition measurements [7,10-14].

Until now, bioelectrical impedance analysis (BIA) has been considered as the simplest, most reproducible and least expensive method for body composition evaluation in clinical practice, and it showed high accuracy and excellent correlation with dual-energy X-ray absorptiometry(DXA) in assessing BF% [15-17]. Therefore, BIA is considered the most cost-effective and feasible replacement for DXA in assessing body composition. BF% has been most commonly used in practice. However, the accuracy of BF% measurements is dependent on height and cannot be evaluated independently from fat free mass (FFM) [18]. Moreover, percentage of body fat does not adjust appropriately for body size, although height has recently been reported as an independent risk factor for CVD [19]. Therefore, both fat mass and fat-free mass should be normalized for body size [20]. Acknowledging such a problem, VanItallie et al. proposed a fat-free mass index (FFMI; FFM⁄ height^2^) and a fat mass index (FMI; FMI ⁄ height^2^) that considers an individual’s height [21]. FMI and FFMI are calculated by dividing fat mass (FM) and fat-free mass (FFM) by the square of height. These calculated body fat indices eliminate the differences of the BF% associated with one’s height, can independently evaluate body fatness from changes in FFM, and therefore, could be a useful measure of obesity [22-24].

The objective of this study was to investigate the best indicator to predict the presence of MetS among fat mass index, BMI and percentage of body fat (BF%) and determine its optimal cut-off value in the screening of MetS in practice.

## Methods

Subjects in the study were enrolled from 2179 people who participated in the annual health check-up in the Department of Medical examination center, Peking Union Medical College Hospital, China Academic Medical Science and Peking Union Medical College, China, in 2011(from January to July). A standard questionnaire was used by trained physicians to collect related information including age, sex, physical activity, smoking, and medication use, and then routine physical examinations were performed to all subjects, and two blood pressure recordings were obtained from the right arm of subjects in a sitting position after 30 min of rest; Diabetes or hypertension were recorded if a participant gave a positive answer to the question: Have you ever been diagnosed by a physician as diabetes or hypertension? Smoking status was categorized as current smokers and non-smokers (nonsmokers or stop smoking for at least 6 months). People who exercised three or more times a week for >30 minutes were categorized as the regular exercise group, and those who exercised less than three times a week were considered the non-regular group. The exclusion criteria were as the follows: 1) with the evidence of cancer, renal, or hematological disease; 2) a medication history of corticosteroids in the previous 6 months; 3) those who were going on a weight-loss program or weight loss ≥5% of body weight within 12 months; 4) people who refused to participate in this study.

Finally, 1698 subjects aged 20 ~ 79 years were enrolled in the study (1105 men and 593 women). All of the study procedures were approved by the Ethics Committee of Peking Union Medical College Hospital, China Academic Medical Science. All subjects provided informed consent to participate in the study.

### Anthropometric measurements

Anthropometric measurements of individuals wearing light clothing and without shoes were conducted by well-trained examiners. Height was measured to the nearest 0.1 cm with a portable stadiometer. Weight was measured in an upright position to the nearest 0.1 kg with a calibrated scale. Body mass index was calculated by dividing weight (kg) by height squared (m^2^). Waist circumference measurements were taken at the end of normal expiration to the nearest 0.1 cm, measuring from the midway between the lower borders of the rib cage and the iliac crest.

### Body composition measurements

Fat mass, percent body fat were measured by multi-frequency bioelectric impedance analysis (multi-frequency bioelectric impedance analyzer Inbody 720, 8 contact point, 5, 50, 250, and 500 kHz,Biospace Co. Ltd., Seoul, Korea). Four electrodes were placed on the palm and thumb of both hands, and four on the anterior and posterior aspects of the soles of both feet. Then fat mass index was calculated by dividing the each subject’s fat mass (kg) by square of his/her height (m). FMI levels were divided into separate quartiles for men and women. Sex-specific FMI quartiles were used as follows: Q1: <4.39, Q2: 4.39- < 5.65, Q3: 5.65- < 7.03, Q4: ≥7.03, in men; and Q1: <5.25, Q2: 5.25- < 6.33, Q3: 6.33- < 7.93, Q4: ≥7.93, in women.

### Biochemical measurements

Blood sample were collected from the subjects’ peripheral vein in the morning after a fasting period of 10–12 h. the samples were immediately centrifuged at 4°C, and plasma for assays of lipid profile (including total cholesterol (TC), triglyceride (TG), low-density-lipoprotein cholesterol (LDL-C), and high-density-lipoprotein cholesterol (HDL-C)), fasting blood glucose (FBG), C-reactive protein (CRP), using an automated analyzer (Olympus AU5400, Japan).

### Definition of metabolic syndrome and overweight/obesity

The MetS was defined using the updated National Cholesterol Education Program/Adult Treatment Panel III criteria (NCEP-ATP III) [25] for Asian Americans as having ≥3 of the following components: waist circumference ≥ 90 cm for men or ≥80 cm for women; triglycerides ≥1.7 mmol/L; HDL cholesterol <1.03 mmol/L for men or <1.30 mmol/L for women; blood pressure ≥130/85 mm Hg or current use of antihypertensive medications; or fasting glucose ≥5.6 mmol/L, type 2 diabetes mellitus previously diagnosed by a physician, or current use of antidiabetic medications. Overweight and obesity were defined as a participant with body mass index (BMI) ≥24 and <28 kg/m^2^, and BMI ≥28 kg/m^2^ respectively, according to the cut-off point [26] for Chinese adults, or BF% ≥ 25% (men) and BF% ≥ 30% (women).

### Statistical analysis

Statistical analysis were performed separately according to sex by using the Statistical Package for Social Science (SPSS version 11.5, Chicago, IL, USA) continuous variables were expressed as means ± SD, whereas categorical variables were represented by frequency and percentage. The differences between two sexes and two groups (Mets group and non- Mets group) were examined by t-test for continuous variables and by *X*^*2*^ test for categorical variables, respectively. The association between the sex-specific fat mass index quartile and metabolic syndrome were tested using Binary Logistic regression analysis, and we calculated the unadjusted and adjusted odds ratio (ORs) using the lowest quartile as the reference. Receiver operating curve (ROC) analysis were used to determine optimal cutoff points for BMI, FMI and BF% in relation to the area under the curve (AUC), sensitivity and specificity in men and women. The values of FMI, BMI and BF% that resulted in maximizing the Youden index (sensitivity + specificity-1) were defined as optimal. P < 0.05 was considered significant for all the statistical analysis.

## Results

The characteristics of the 1698 participants are summarized in Table 1. In this study population, 232 men (21.00%) and 109 women (18.40%) were diagnosed with MetS by NCEP-ATP III criteria. The mean of many parameters (including BMI, WC, DBP, TC, TG, LDL, FBG, CRP) and the percentage of smokers were significantly higher in men than in women (*P* < 0.001), but the mean HDL, BF%, FMI, and the percentage of regular exerciser were significantly lower in men than in women (*P* < 0.001). No significant differences in the mean of age or SBP and in the prevalence of MetS were found between men and women.

**Table 1:** The general characteristics according to sex

**Variables**	**Men(n = 1105)**	**Women(n = 593)**	***P *value**
Age(yr)	46.96 ± 8.46	46.45 ± 10.79	0.319
Body mass index(kg/m2)	26.01 ± 3.02	23.31 ± 3.36	0.000
Fat mass index(kg/m2)	5.78 ± 2.12	6.74 ± 2.25	0.000
Percentage of body fat(%)	21.59 ± 6.11	28.11 ± 5.91	0.000
Waist circumference(cm)	84.67 ± 7.35	77.81 ± 6.60	0.000
Systolic blood pressure(mmHg)	124.24 ± 17.84	124.83 ± 21.63	0.565
Diastolic blood pressure(mmHg)	77.34 ± 11.95	73.17 ± 11.1	0.000
Total cholesterol(mmol/L)	4.95 ± 0.91	4.79 ± 0.90	0.001
Triglyceride(mmol/L)	2.03 ± 1.43	1.29 ± 0.90	0.000
High-density-lipoprotein cholesterol(mmol/L)	1.12 ± 0.25	1.38 ± 0.31	0.000
Low-density-lipoprotein cholesterol(mmol/L)	3.12 ± 0.79	2.94 ± 0.78	0.000
Fasting blood glucose(mmol/L)	5.51 ± 1.25	5.09 ± 0.89	0.000
C-reactive protein(mg/L)	1.84 ± 2.10	1.38 ± 1.83	0.000
Prevalence of MetS (%/n)	21.00/232	18.40/109	0.200
Percentage of Smokers (%/n)	38.40/424	19.2/114	0.000
Percentage of regular exerciser (%/n)	32.3/357	42.7/253	0.000

In MetS group for both sexes (Table 2), variables including BMI, WC, WHtR, WHR, BF%, FMI, SBP, DBP, TG, FBG, and CRP were significantly higher than those in non- MetS group (*P* < 0.001), but HDL was significantly lower than that in non- MetS group (*P* < 0.001). No significant differences in the mean of TC or the percentage of current smokers were found between MetS group and non- MetS group for both sexes. In addition, percentage of regular exercise (*P* = 0.008) and mean LDL (*P* = 0.007) in MetS group of men were significantly lower than in non- MetS group, and mean age in MetS group of women was higher than in non- MetS group (*P* < 0.001).

**Table 2:** The descriptive characteristics of participantsts with and without MetS in both sexes

**Men**				**Women**		
**Variables**	**MetS**	**non-MetS**	***P *value**	**MetS**	**non-MetS**	***P *value**
Age(yr)	46.98 ± 7.38	46.96 ± 8.73	0.971	52.25 ± 9.56	45.15 ± 10.63	0.000
BMI(kg/m^2^)	29.49 ± 2.59	25.09 ± 2.39	0.000	27.33 ± 3.38	22.41 ± 2.61	0.000
WC(cm)	93.84 ± 6.28	82.23 ± 5.43	0.000	85.94 ± 6.68	75.98 ± 5.02	0.000
BF(%)	27.88 ± 4.67	19.92 ± 5.31	0.000	34.11 ± 4.58	26.76 ± 5.30	0.000
FMI(kg/m^2^)	8.33 ± 1.87	5.10 ± 1.60	0.000	9.46 ± 2.22	6.12 ± 1.75	0.000
SBP(mmHg)	129.15 ± 17.76	122.93 ± 17.64	0.000	138.82 ± 20.65	121.69 ± 20.60	0.000
DBP(mmHg)	84.00 ± 11.48	75.57 ± 11.45	0.000	79.72 ± 12.39	71.69 ± 10.36	0.000
TC(mmol/L)	4.94 ± 0.91	4.95 ± 0.91	0.921	4.92 ± 1.01	4.76 ± 0.87	0.118
TG(mmol/L)	3.19 ± 2.21	1.73 ± 0.98	0.000	2.24 ± 1.36	1.08 ± 0.58	0.000
HDL(mmol/L)	0.99 ± 0.21	1.16 ± 0.25	0.000	1.12 ± 0.22	1.44 ± 0.30	0.000
LDL(mmol/L)	2.99 ± 0.89	3.16 ± 0.76	0.008	3.03 ± 0.83	2.93 ± 0.76	0.238
FBG((mmol/L))	6.42 ± 1.77	5.27 ± 0.93	0.000	5.84 ± 1.67	4.92 ± 0.43	0.000
CRP(mg/L)	2.60 ± 2.37	1.64 ± 1.98	0.000	2.02 ± 1.88	1.23 ± 1.79	0.000
Current smokers(%)	40.10	37.90	0.545	21.1	18.8	0.582
Regular exercise(%)	25.00	34.20	0.007	45.9	41.9	0.454

### The prevalence of MetS according to FMI quartiles

**Figure 1:**
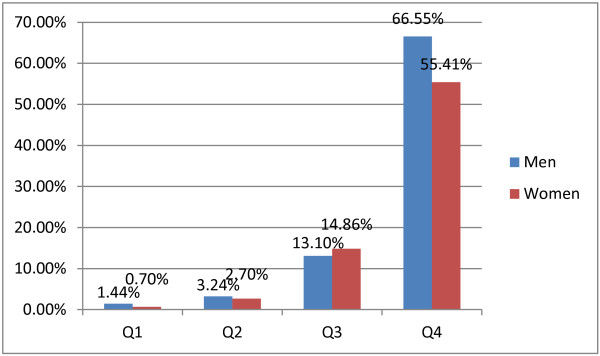
Prevalence of metabolic syndrome (MetS) according to the FMI quartiles.

The prevalence of MetS in each FMI quartile level were 1.44%, 3.24%, 13.10% and 66.55% for men, and 0.70%, 2.70%, 14.86 and 55.41 for women, respectively. The prevalence of MetS significantly increased in 3^rd^ and 4^th^ FMI quartiles for both men and women (P < 0.001 for both sexes) (Figure 1).

### Odds ratio and 95% confidence intervals for the presence of MetS using the lowest FMI quartile as the reference

Table 3 shows the risk of MetS according to the FMI quartiles. After adjustment for age, BMI, BF%, TC, LDL, CRP, smoking status and exercise status, the 3rd and 4th FMI quartiles had significantly higher Odds ratio for metabolic syndrome than the lowest quartile in both sexes. The adjusted OR (95% CI) for the presence of metabolic syndrome in subjects with the highest FMI quartile was 52.039 (4.144-653.436) for women and 79.143 (21.243-294.852) for men, as compared to the subjects with the lowest FMI quartile. When WC was added as a additional adjusted factor into regression analysis, the 3^rd^ and 4^th^ quartiles of FMI for men and the 4^th^ quartile of FMI for women also had significantly higher Odds ratio for metabolic syndrome than the lowest quartile.

**Table 3:** **Odds ratio and 95**% **confidence intervals for the presence of MetS according to the FMI quartiles in men and women**

**Odds ratio(95% confidence interval)**				
**Gender**	**FMI(kg/m**^**2**^**)**	**Unadjusted**	Adjusted*	**Adjusted $**
Women	Quartile 1(<5.25)	1	1	1
	Quartile 2(5.25-6.33)	4.083(0.451-36.975)	2.722(0.288-25.749)	3.127(0.340-30.464)
	Quartile 3(6.33-7.93)	25.465(3.385-191.583)^	10.584(1.190-94.111)#	8.397(0.917-76.883)
	Quartile 4(>7.93)	182.636(24.887-1340.296)^	52.039(4.144-653.436)&	25.574(1.945-336.228)#
Men	Quartile 1(<4.39)	1	1	1
	Quartile 2(4.39-5.65)	2.283(0.695-7.504)	1.329(0.380-4.650)	1.305(0.372-4.584)
	Quartile 3(5.65-7.03)	10.280(3.607-29.304)^	4.698(1.480-14.909)#	3.860(1.203-12.392)#
	Quartile 4(>7.03)	135.758(49.031-375.890)^	79.143(21.243-294.852)&	9.166(2.157-38.952)&

*Adjusted for age, smoking status, exercise status, total cholesterol, low-density-lipoprotein cholesterol, percentage of body fat and C-reactive protein. $ added WC into * model; # *P* < 0.05; &*P* < 0.01; ^ *P* < 0.001.

### Other parameters’ odds ratio and 95% confidence intervals for the presence of MetS using the lowest FMI quartile as the reference

According to Binary logistic regression analysis, besides high FMI level, overall overweight or obesity, and elevated CRP level, were independently associated with MetS in men (X^2^ = 51.032, *P* < 0.001), whereas in women subjects, age, overall overweight or obesity, and elevated CRP level were independently associated with MetS (X^2^ = 36.327, *P* < 0.001). Odds ratio of MetS in both sexes were shown in Table 4.

**Table 4:** **Parameters**’ **Odds ratio of MetS in both sexes using the lowest quartile as the reference**

**Men**				**Women**		
**Disorder**	wald *X*^*2*^	**OR(95% CI)**	***p *value**	**wald *X***^***2***^	**OR(95% CI)**	***p* value**
Age	0.142	0.920(0.595-1.422)	0.706	10.666	2.701(1.488-4.903)	0.001
BMI (≥24)	3.951	4.783(1.022-22.381)	0.047	9.854	4.082(1.696-9.822)	0.002
BF% (>25 or 30)	0.149	0.871(0.431-1.760)	0.700	1.037	0.570(0.193-1.682)	0.308
TC (≥5.71)	0.051	1.060(0.638-1.762)	0.822	0.039	0.917(0.388-2.165)	0.843
LDL (≥4.14)	3.590	0.517(0.261-1.023)	0.058	0.676	0.595(0.173-2.051)	0.411
CRP (quartile)	40.434	1.844(1.527-2.227)	0.000	25.384	2.084(1.566-2.774)	0.000
Exercise status	0.494	0.849(0.538-1.341)	0.482	3.655	1.835(0.985-3.419)	0.056
Smoking status	0.012	0.978(0.649-1.473)	0.914	2.345	1.819(0.846-3.914)	0.126

### ROC curve analysis of MetS-associated indicators to predict MetS

The areas under ROC curve, the cutoff values, and the most appropriate sensitivities and specificities of the indicators are presented in Table 5.

**Table 5:** **Sensitivity**, **specificity and AUC of cutoff value of three indicators in prediction of MetS**

**Indicators**	**Cutoff value**	**Sensitivity**	**Specificity**	**AUC**	**95%CI**	**P value**
BMI(kg/m^2^)						
Men	27.45	0.806	0.843	0.904	0.882 ~ 0.925	0.000
Women	23.85	0.927	0.729	0.898	0.869 ~ 0.928	0.000
BF(%)						
Men	23.95	0.841	0.778	0.883	0.859 ~ 0.908	0.000
Women	31.35	0.771	0.814	0.855	0.818 ~ 0.892	0.000
FMI(kg/m^2^)						
Men	7.00	0.802	0.869	0.920	0.900 ~ 0.940	0.000
Women	7.90	0.789	0.857	0.898	0.869 ~ 0.927	0.000

**Figure 2:**
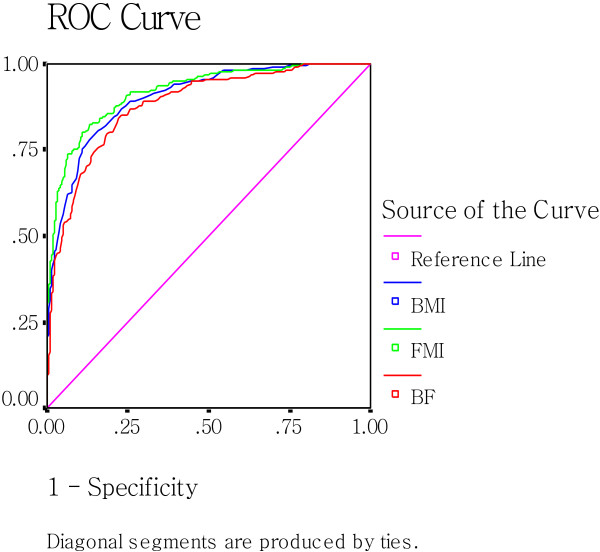
Receiver-operating characteristic(ROC) analysis of BMI, BF%, and FMI as indicators to predict MetS in men.

**Figure 3:**
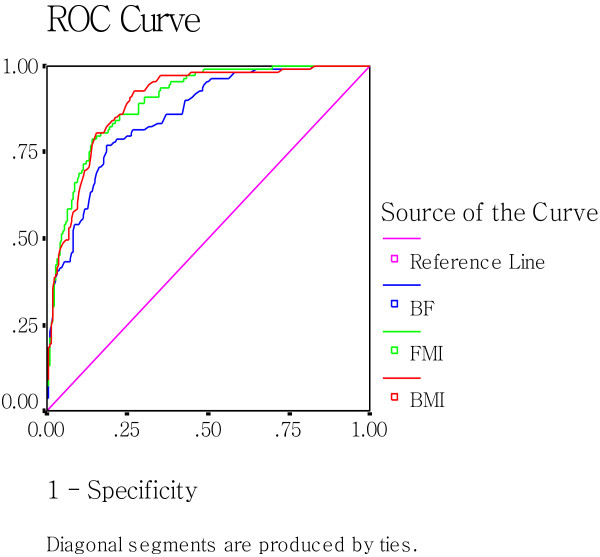
Receiver-operating characteristic(ROC) analysis of BMI, BF%, and FMI as indicators to predict MetS in women.

As shown in Figure 2 and 3, which include the ROC curves of BMI, BF%, and FMI, It can be observed that the line referring to the FMI possesses the largest projection for the upper left corner of the curve in the three parameters in both sexes (AUC_FMI_ = AUC_BMI_, in women), which indicates its best predictive potential among the the parameters.

## Discussion

Metabolic syndrome is associated with the development of diabetes, cardiovascular disease, which is the leading cause of mortality worldwide [27] and epidemical in China and other economically developing countries in recent decades [28]. In addition, MetS was associated with arteral stiffness, which was a cardiovascular outcome of MetS [29]. Therefore, it is very important to develop an effective screening tool of metabolic syndrome in practice in China.

To our best knowledge, this is the first large cross-sectional study that examined the association of fat mass index quartiles (by BIA) and metabolic syndrome and determine the optimal cut-off values of fat mass index in prediction of metabolic syndrome in practice in Chinese population.

In this study, BMI, BF% and FMI were used to screen the presence of metabolic syndrome. One study [30] concluded that the BMI, waist circumference and waist-to-height ratio can predict the presence of multiple metabolic risk factors in Chinese subjects, but parameters including WC were not used in this study, since WC is a part of the definition of metabolic syndrome. BMI is an anthropometric parameter which is widely used to the assessment of obesity, and it is easily calculated. However, it cannot reflect body fat mass and body fat distribution due to the differences of age, sex and ethnic groups and obese types when BMI is used alone. Although some studies [10,31] found that high BF% was associated with increased cardiovascular risk regardless of BMI whose categorization resulted in an underestimation of subjects with cardiovascular risk factors [32], people with the same BMI or the same percentage may have very different body composition, which may result in people with the same BMI or percentage of body fat exposing to different metabolic conditions, therefore, it is better to measure and express body composition as FMI and FFMI than either BMI or BF% [33]. Among the indicators used to predict the presence of metabolic syndrome, FMI was the index that showed the greatest area under the ROC curve. In addition,our study showed that high FMI had significantly higher Odds ratio for metabolic syndrome than the low FMI in both sexes, which was similar to one previous study [33], in which body composition was measured by DXA. Our study also showed that high FMI level was strongly associated with the presence of MetS after adjusting BMI and BF% in both men and women, and the adjusted odds ratios of the risk of MetS were higher than that of BMI and BF%. Moreover, when WC was also added into regression analysis the 3^rd^ and 4^th^ quartiles of FMI for men and the 4^th^ quartile of FMI for women still had significantly higher Odds ratio for metabolic syndrome than the lowest quartile.

Therefore,our study shows that high FMI level appears to be independently associated with MetS regardless of BMI and BF% and can be the effective measurement method for the assessment of metabolic syndrome in clinical practice. FMI of 7.00 kg/m^2^ for men and 7.90 kg/m^2^ for women by BIA could effectively predict the presence of MetS in our study. A recent study [34] showed that waist-to-height ratio, which was a better screening tool than BMI and waist circumference for adult metabolic risk factors demonstrated in a recent systematic review [35], was the best screening tool for evaluation MetS in Korean men, and adding FMI could result in a modest increase in integrated discrimination improvement. Data analysis for the combination of waist-to-height ratio with FMI are not shown in this study, further investigation should be conducted with the aim at determining whether the combination is appropriate for Chinese population.

In addition, our study determined that the CRP level were significantly higher in postmenopausal women with MetS than those without MetS and higher CRP level is independent factor for the presence of metabolic syndrome, which is consistent with one recent study [36]. In addition, the mean age was significantly higher in MetS group than in non-MetS group (52.25 ± 9.56 vs. 45.15 ± 10.63, P < 0.001) and age seemed to be one of the risk factors of the presence of MetS in women, the possible reason was that postmenopausal women were occupational in the MetS group, and postmenopausal women are likely to have the increasing prevalence of insulin resistance and obesity (particularly visceral adiposity), which might contribute to the risk of MetS [14].

This study has several limitations. First, due to the cross-sectional design, it is not possible to explore the causal relationship between body composition and metabolic syndrome. Second, our population is restricted to 20-79-year-old men and women living in Beijing,and the result from oue study might not be applicable to other population. Third, hypertriglyceridemia, Low HDL-c, fasting hyperglucemia did not be included in the regression analysis, because they are the essential criteria in the diagnosis for MetS. Fourth, the prevalence of metabolic syndrome with age group (particularly in women since the post-menopause status might contribute to the risk of MetS due to the excessive fat accumulation) was not mentioned in this article, and further investigation should be performed in the future studies.

## Conclusion

Despite these limitations, our study suggested that FMI seems to be a better indicator in the screening of the presence of metabolic syndrome than BMI and percentage of body fat in men and women.

## Abbreviations

MetS: Metabolic syndrome; BMI: Body mass index; WC: Waist circumference; SBP: Systolic blood pressure; DBP: Diastolic blood pressure; TC: Total cholesterol; TG: Triglyceride; HDL-C: High-density-lipoprotein cholesterol; LDL-C: Low-density-lipoprotein cholesterol; FBG: Fasting blood glucose; CRP: C-reactive protein; BF%: Percentage of body fat; FMI: Fat mass index.

## Competing interests

The authors declare that they have no competing interests.

## Authors’ contributions

MF was responsible for the study design. LPJ participated in the design of the study and drafted the manuscript. LHP participated in the sequence alignment. LYP performed the statistical analysis. All authors read and approved the final manuscript.

## Pre-publication history

The pre-publication history for this paper can be accessed here:

http://www.biomedcentral.com/1471-2458/13/629/prepub
